# Optimization of Anti-Fouling Piezoelectric Composite Coating for High-Voltage Insulators in Converter Stations

**DOI:** 10.3390/ma18235270

**Published:** 2025-11-21

**Authors:** Yanwen Ouyang, Meng Chen, Siwei Pan, Qing Wang, Yihua Qian, Yuanyuan Li, Yong Liu, Pengfei Fang

**Affiliations:** 1 School of Physics and Technology, Wuhan University, Wuhan 430072, China; 2019102020078@whu.edu.cn (Y.O.);; 2 Hubei Key Laboratory of Marine Electromagnetic Detection and Control, Wuhan 430064, China; 3 Wuhan Second Ship Design and Research Institute, Wuhan 430064, China; 4 Electric Power Research Institute of Guangdong Power Grid Co., Ltd., Guangzhou 510080, China

**Keywords:** room-temperature vulcanizing, BaTiO_3_, insulator anti-fouling, DC field contamination accumulation

## Abstract

Under the DC field, live contamination is more likely to deposit on the surface of insulators due to the action of the external electric field. The deposition of dirt on the surface of Ultra High Voltage (UHV) insulators can lead to the occurrence of flashover phenomena, causing significant economic losses. Due to the particularity of UHV insulators, many traditional surface anti-pollution technologies designed for normal voltage insulators are not applicable to them. In order to prevent the harm of contamination accumulation affecting the safe operation of transmission lines, in this study, tetragonal BaTiO_3_ was mixed into room-temperature vulcanized silicone rubber for the first time to prepare a composite coating with piezoelectric properties. This coating can use the piezoelectric effect to remove the contamination adhering to the surface of UHV insulators under a DC field. In this study, the piezoelectric properties of the prepared tetragonal BaTiO_3_ were verified through material characterization. The results show that the introduction of piezoelectric fillers can significantly accelerate the dissipation of charges on the insulator surface under slight disturbances, which helps to reduce the accumulation of charged pollutants on the insulator surface. The anti-pollution performance under electric field conditions was verified through a simulation experimental device. Finally, through experiments in a real converter station environment, the anti-pollution effect of the insulator under actual working conditions was verified.

## 1. Introduction

Pollution not only reduces the quality of life of people but also affects the normal operation of the power system [1,2,3,4]. Under normal circumstances, to ensure the safe operation of the power system, the surface of insulators usually has sufficient insulation redundancy. However, outdoor insulators are naturally affected by various pollutants. When pollutants adhere to the surface of the insulator, in wet weather conditions such as rain, fog, and dew, the surface will be fully wetted to form a conductive area, thereby significantly reducing the surface resistance of the insulator [5,6,7]. In this case, the possibility of contamination flashover of the insulator increases significantly.

The relationship between pollution and flashover is well documented. The levels of insulator pollution, measured by parameters such as Equivalent Salt Deposit Density (ESDD) and Non-Soluble Deposit Density (NSDD), reflect the probability of flashover occurrence [7,8]. Experiments show that ESDD is directly related to surface conductivity. As ESDD increases, the surface conductivity also increases [9,10,11,12]. NSDD significantly affects the surface wettability of insulators. High NSDD enhances surface wettability, promoting the dissolution of ESDD, which in turn increases the electrical conductivity of the insulator surface [13]. Studies have shown that, compared with non-energized insulators, energized insulators tend to accumulate more pollution, which means higher ESDD and NSDD [14]. The lower surface of charged composite insulators has significantly higher ESDD and NSDD compared to uncharged ones [15]. The motion of charged particles is also affected by various forces, including electric field force, polarization force, resistance, and gravitational force. Among them, the electric field force plays a dominant role [16,17,18]. Under a direct current (DC) electric field, compared with an alternating current (AC) electric field, pollution accumulation is more obvious [19,20,21]. This is because in a DC field, the electric force changes the vertical movement speed of particles and accelerates their deposition process [22,23,24].

Regular maintenance practices, including cleaning and recoating insulators, are crucial for managing pollution and ensuring the service life of insulators. However, a large amount of cost is incurred annually for cleaning and maintenance [25,26]. To address the flashover problem caused by pollution accumulation on insulators, a lot of research has been conducted. For example, Room Temperature Vulcanized (RTV), because of its high adhesion and high insulation properties to insulators, is usually used as a coating to improve the insulation and anti-pollution ability of the insulator surface. However, the insulating fillers added to the RTV coating will introduce a large number of deep traps, which will lock the internal charges of the insulator, inhibit the migration of surface charges, and lead to the continuous accumulation of surface charges. Piezoelectric fillers have the ability to regulate surface charges and enhance charge migration [27,28,29,30]. Kwon et al. introduced cubic and tetragonal ${\mathrm{BaTiO}}_{3}$ into PDMS to regulate surface charges [31]. Wang et al. enhanced the charge migration ability by introducing (${\mathrm{Ba}}_{0.838}$${\mathrm{Ca}}_{0.162}$)(${\mathrm{Ti}}_{0.9072}$${\mathrm{Zr}}_{0.092}$)O_3_ powder through a dielectrophoresis strategy [32]. These examples indicate that piezoelectric fillers have application prospects in surface charge regulation.

In this study, based on the need for UHV anti-fouling, RTV was used as the base material, and barium titanate (BTO) piezoelectric fillers were introduced. Through the slight disturbances in the external environment (such as the mechanical stress generated by vibrations caused by wind and rain), dynamic and controllable traps were introduced into the RTV. This promotes the migration of surface charges to the inside, thereby accelerating the dissipation of surface charges on the insulator, further reducing the deposition of charged particles in the electric field, and reducing the probability of pollution flashover of the insulator. This study provides ideas for the anti-pollution work of insulators and can be used to maintain the safe operation of the power grid.

## 2. Materials and Methods

### 2.1. Experimental Materials

Polydimethylsiloxane (PDMS) was purchased from Dow Corning 184, Dow Inc., Midland, MI, USA. Titanium dioxide (P25, purity 99.7%) was purchased from Evonik Industries AG, Hanau, Germany. Dopamine hydrochloride(PDA, purity 98%) and Tris-Hcl (purity 99.9%) were purchased from Aladdin Biochemical Technology Co., Ltd., Shanghai, China. Fumed silica (974, purity 99.8%) was purchased from Evonik Industries AG, Germany. Iron oxide red (H130, purity 95%) was purchased from Three-Ring Pigments, Changsha, China. Another titanium dioxide (R-706, purity 96%) was purchased from DuPont, Wilmington, DE, USA. All other chemical reagents used in the experiment were provided by Sinopharm Chemical Reagent Co., Ltd., Shanghai, China.

### 2.2. Preparation of Coating

BTO preparation: P25 (1.0 g) is dispersed in 50 mL of 8 M NaOH solution, followed by ultrasonic dispersion for 30 min. Add 12.5 mL of 1 M ${\mathrm{BaCl}}_{2}$ solution to the mixture and stir for 3 h. Pour the mixture into a reaction kettle and conduct a hydrothermal reaction at 180 °C for 24 h. Collect the precipitate. Finally, wash away the residual ${\mathrm{Na}}^{+}$ ions with deionized water until the solution is neutral. After drying, BTO is obtained. Add 0.2422 g Tris-HCl into 200 mL deionized water, adjust the pH to 8.5. A total of 0.2 g dopamine hydrochloride was dissolved in 50 mL of the buffer with pH maintained at 8.5. 1 g Pre-synthesized BTO nanoparticles were added gradually to the solution. The mixture was sonicated for 15 min and continuously stirred at 500 rpm and 25 °C for 24 h. The particles were collected by centrifugation for 15 min, washed three times with deionized water, and vacuum-dried at 60 °C for 12 h to obtain PDA-coated BTO powder.

Coating preparation: Propylene glycol methyl ether acetate and n-Butanol are mixed in a 5:7.5 ratio and poured into a container. Stir with a high-speed mixer for 15 min. Pour 77 g of PDMS into the mixture and stir for 30 min. Add 1.3 g of aluminum hydroxide, 1.7 g of silicon dioxide, and 4 g of BTO, and stir for 30 min. Then, if necessary, add an appropriate amount of H130 (denoted as pRTV-R) or R-706 (denoted as pRTV-W) as a coloring agent and sonicate for 1 h to obtain Component A. Weigh 10 g of n-butanol and mix it uniformly with 10 g of TEOS, and stir for 30 min. Then add an organotin catalyst and stir for 20 min. Filter the mixture and store it in a sealed container, denoted as Component B.

Spraying insulator samples: During sample preparation, the surface area of the insulator is estimated and calculated. Take out and weigh the prepared coating components at a dosage of $1.0\pm 0.1\;\mathrm{kg}/10\;m^{2}$. Mix the components evenly according to the ratio of Component A:Component B = 9:1, pour them into a spray bottle, stir well, and let it stand for 10 min. Adjust the pressure of the spraying machine to 0.8±0.1MPa, and spray the insulator samples. Spray one side a total of three times, with an interval of 20 min between each spraying. Finally, a coating material with a dry film thickness of about 100±20 µm is obtained.

### 2.3. Testing and Characterization

#### 2.3.1. Characterization

The surface morphologies were characterized by scanning electron microscopy (JEM-2100Plus, FEI, JEOL Ltd., Tokyo, Japan) and transmission electron microscopy (JEM-2100 FEF, JEOL). The composite’s piezoelectric response was quantified via the output performance test of a piezoelectric nanogenerator (PENG). The piezoelectric voltage was recorded by a digital oscilloscope (DS2202A, Rigol Technologies, Suzhou, China). The electrostatic properties were measured using an electrostatic measuring instrument (FMX-03, Schneider, Singapore). The surface resistance of the coating was measured using a surface resistance meter (MODEL-100, Hazfull, Singapore). To identify the crystalline phases, X-ray diffraction (Smartlab, Rigaku, Tokyo, Japan) with Cu Kα radiation was utilized. The contact angle meter (SL200B, Kono, London, UK) was used to measure the water contact angle. The conductivity tester (DDS-11A, YUEPIN, Foshan, China) was used to measure the conductivity of the liquid.

#### 2.3.2. Insulator Surface Potential Measurement

Samples were prepared using the coating preparation method described in Section 2.2 and applied to the cut insulator sheds (model: FXBW4-10/70, Nanjing Gulifa Electric Co., Ltd., Nanjing, China). Among them, the blank insulator was denoted as Blank, the insulator coated with pure PDMS was denoted as RTV, and the insulator coated with BTO filler was denoted as pRTV. A needle electrode was used to polarize the surface of the insulator samples. The distance between the needle tip and the shed surface was 20 mm, the applied voltage was 7 kV, and the discharge time was 15 min. As shown in Figure S1b, immediately after the polarization treatment was completed, 8 points were evenly selected on the surface of the composite insulator sample, and an electrostatic measuring instrument was used to measure the surface voltage distribution in sequence and record the results.

#### 2.3.3. Equivalent Salt Deposit Density and Non-Soluble Deposit Density

Equivalent salt deposit density (ESDD) refers to the amount of NaCl ($\mathrm{mg}/{\mathrm{cm}}^{2}$) equivalent to the content of conductive substances in the pollution adhering to each square centimeter of the insulator surface. Non-soluble deposit density (NSDD) refers to the mass of insoluble substances ($\mathrm{mg}/{\mathrm{cm}}^{2}$) adhering to each square centimeter of the insulator surface.

Before collecting pollution samples, measure and record the surface area of the insulator shed. When collecting filthy samples, use absorbent cotton wetted with deionized water to carefully wipe the upper and lower surfaces of the insulator shed. Use one piece of absorbent cotton for each shed to collect the samples. Then, stir the absorbent cotton with 500 mL of deionized water to disperse the soluble and insoluble substances on the absorbent cotton. Stir for 2 h and let it stand for 4 h. Use a conductivity tester to measure and record the conductivity of the liquid, and then calculate the ESDD of the pollutants according to the formula. Weigh and record the weight of the filter paper. Use a Buchner funnel and a vacuum pump to filter the stirred liquid to collect the insoluble substances. Then weigh and calculate the weight of the filter paper after filtration, and calculate the NSDD of the pollutants according to the formula. See the Supplementary Materials for the calculation method.

#### 2.3.4. Insulator Pollution Accumulation Simulation

To characterize the surface contamination performance of coated specimens, an insulator fouling simulation apparatus was employed as shown in Figure S2. This experimental system comprises four integrated modules: (1) a high-voltage power supply unit, (2) an axial-flow air propulsion system with adjustable wind velocity, (3) a particulate dispersion mechanism containing contamination mixtures, and (4) a rotary stage enabling controlled azimuthal rotation. The high-voltage module establishes electrostatic field conditions mimicking operational environments. Concurrently, the aerodynamic and particulate delivery subsystems replicate atmospheric deposition processes. The electromechanical rotation assembly ensures that pollutants are evenly distributed on the surface of the insulator at a controllable rotational speed during the test.

Before the experiment, the insulator surfaces were wiped with alcohol and air-dried in a shaded environment. During testing, the insulator was fixed onto the rotary stage and set to rotate at 20 rpm. The test chamber was closed, and the axial-flow air propulsion system was activated to adjust the wind speed to 20 m/s. The high-voltage power supply was then turned on, and the insulator was pre-charged for 15 min. Subsequently, 20 g of kaolin was poured into the pipeline over a 10-minute period. Charging continued for an additional 15 min, after which the high-voltage power supply and the axial-flow air propulsion system were turned off. Surface contaminants were collected from the insulator following the protocol described in Section 2.3.3 and weighed.

## 3. Results and Discussion

### 3.1. Piezoelectric Performance

**Figure 1:**
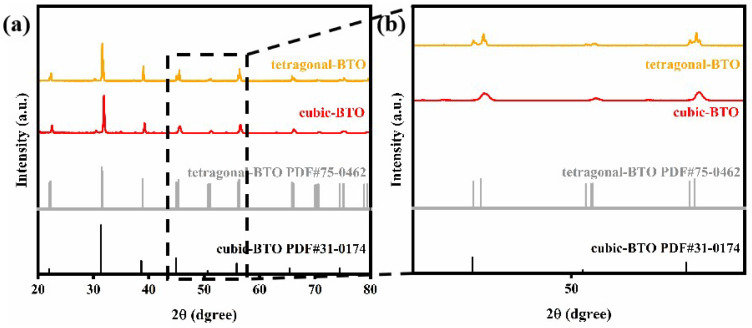
The XRD images show the difference between the cubic-BTO and the prepared tetragonal-BTO in the pRTV. (**a**) XRD images of cubic-BTO and tetragonal-BTO. (**b**) Enlarged XRD images of cubic-BTO and tetragonal-BTO with a 2θ angle between 43° and 57°.

Figure 1a,b shows the XRD pattern of the prepared BTO and its enlarged view. By comparing with the tetragonal-${\mathrm{BaTiO}}_{3}$ JCPDS card 75-0462, it is verified that the prepared material is tetragonal BTO. The enlarged view shows that obvious peak splitting occurs near 45° and 55° in the prepared BTO. The peak splitting at 45° corresponds to the splitting of the (002) and (200) crystal planes of tetragonal BTO, and the peak splitting at 55° corresponds to the splitting of the (211) and (112) crystal planes [33]. The XRD results are consistent with the characteristics of the space group P4mm, which indicates that the prepared BTO has a tetragonal structure. BTO with this structure has a significant piezoelectric effect [34].

**Figure 2:**
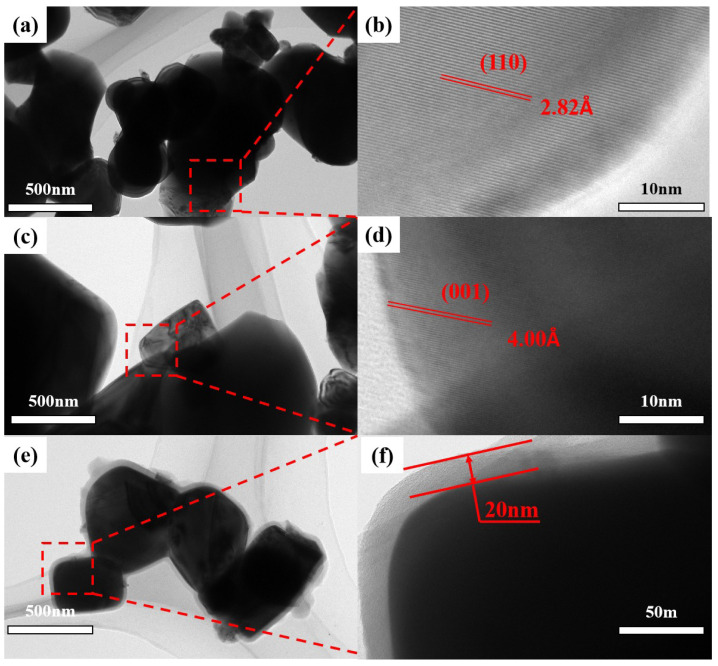
The TEM images of prepared tetragonal-BTO and PDA-coated tetragonal-BTO. (**a**) TEM image of BTO and (**b**) the (110) crystal plane image. (**c**) TEM image of BTO and (**d**) the (001) crystal plane image. (**e**) TEM image of the PDA-coated BTO and (**f**) the magnified view shows that the coating thickness of PDA is 20 nm.

Figure 2a–d are the TEM images of the prepared BTO. Among them, Figure 2a,b show crystal planes with a lattice spacing of about 0.284 nm, and this result corresponds to the (110) crystal plane of tetragonal BTO. Figure 2c,d show crystal planes with a lattice spacing of about 0.389 nm, and this result corresponds to the (100) crystal plane of tetragonal BTO [35]. These two crystal planes are both typical characteristic crystal planes of the tetragonal phase BTO, further proving that the prepared BTO has a tetragonal phase structure with piezoelectric activity. Figure 2e,f are the TEM images of PDA-coated BTO. It can be clearly seen that BTO is coated with a layer of PDA about 20 nm thick, which will contribute to its dispersion in PDMS.

**Figure 3:**
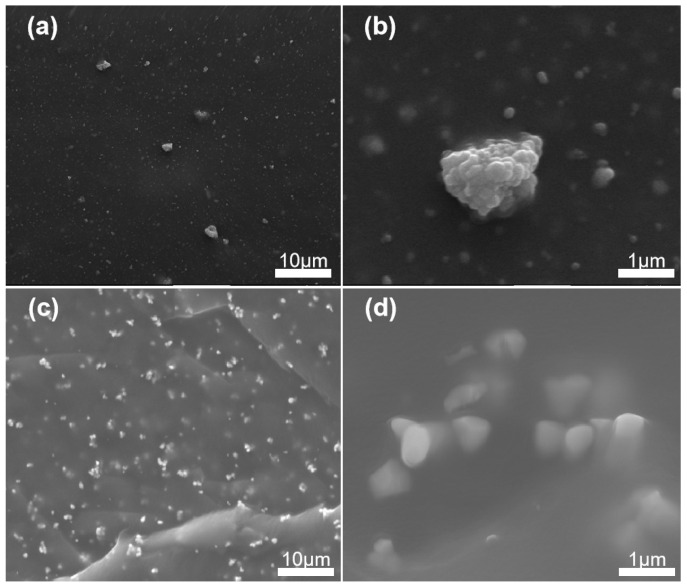
The SEM images of the distribution of BTO in RTV before and after PDA coating. (**a**) Enlarged image of BTO without PDA coating. (**b**) BTO without PDA coating. (**c**) BTO with PDA coating. (**d**) Enlarged image of BTO with PDA coating.

Figure 3 is the SEM image of the coating surface. Figure 3a,b represents the coating with BTO, and Figure 3c,d represents the coating with PDA-coated BTO. It can be seen that BTO is sporadically distributed inside the cross-section of the coating, and agglomeration occurs. This will lead to significant differences in the performance of various parts of the coating, which is unfavorable for the overall piezoelectric effect of the coating. It can be seen that the PDA-coated BTO is widely distributed within the cross-section of the coating without agglomeration. This result indicates that the dispersibility of the BTO is improved, which can effectively enhance the piezoelectric performance of the coating. After PDA coating, the BTO particles no longer agglomerate, and the dispersibility in PDMS is significantly improved. This is because after PDA coating, BTO can form interfacial bonds through surface-active functional groups, which can improve the connection strength between the filler and the polymer matrix interface [36,37].

**Figure 4:**
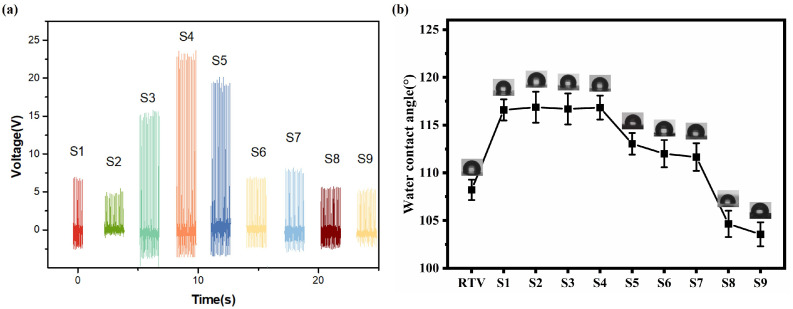
(**a**) Piezoelectric voltages with different BTO ratios (0%, 1.6%, 2.3%, 3.0%, 3.7%, 4.4%, 5.1%, 5.8%, 6.5%, 7.2%). (**b**) Water contact angles with different BTO ratios (0%, 1.6%, 2.3%, 3.0%, 3.7%, 4.4%, 5.1%, 5.8%, 6.5%, 7.2%).

To study the relationship between the piezoelectricity of the coating and the mass fraction of BTO, the piezoelectric electromotive force of the coating at different mass fractions was measured using PENG, as shown in Figure 4a. S1–S9 correspond to samples with gradually increasing mass fractions (1.6%, 2.3%, 3.0%, 3.7%, 4.4%, 5.1%, 5.8%, 6.5%, 7.2%). The results show that the piezoelectric electromotive force of the piezoelectric composite film first increases and then decreases as the composite ratio of the piezoelectric material increases. This is because at the beginning, as the content increases, the piezoelectric potential caused by deformation gradually increases, thereby enhancing the output ability. However, when the content of the piezoelectric material continues to increase, excessive piezoelectric material makes it prone to agglomeration, failing to achieve the ideal arrangement, and thus reducing the contribution of the piezoelectric effect to the composite nanogenerator. Therefore, the 3.7wt% BTO/PDMS composite film was selected for the study of the anti-fouling performance of the insulator surface [38]. As Figure 4b shows, the addition of a small amount of BTO filler will increase the water contact angle, indicating an increase in the hydrophobicity of RTV. When the concentration exceeds 3.7%, the water contact angle will rapidly decrease, indicating a decrease in the hydrophobicity of RTV. The higher concentration causes BTO agglomeration, thereby reducing hydrophobicity [39]. The hydrophobic recovery of the coating after plasma treatment is shown in Figure S3. The tests of the physical, chemical and electrical properties of the coating are shown in Tables S2 and S3.

### 3.2. Coating Surface Characteristics

**Figure 5:**
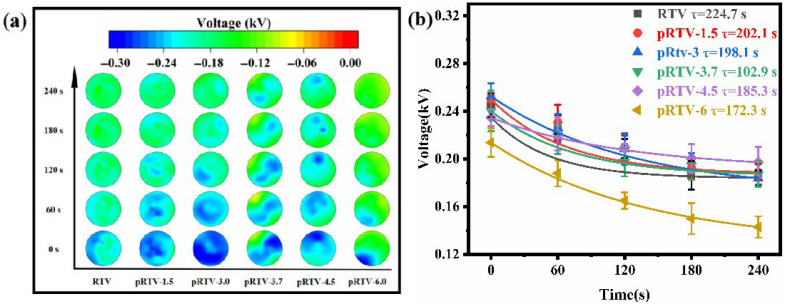
(**a**) Surface potential decay diagrams of six samples after polarization at DC 5 kV for 15 min (measured every 1 min). (**b**) Fitted curves of the average potential decay of the six samples.

Samples of pRTV with five different proportions are shown in Figure S1a. Polarize the samples through a high-voltage power supply. The voltage is DC 5 kV and the pressurization time is 15 min. During the experiment, the initial voltage of each insulator shed surface was measured. Then, the voltage of the insulator shed was measured once every 1 min, for a total of 5 measurements. The measurement results of the surface voltage are shown in Figure 5a. Figure 5b shows the average voltage reduction curve. The decay of the surface potential with time *t* follows an exponential function, where τ represents the dissipation rate of the surface charge [40,41].

(1) $$V\left(t\right)=V_{0}e^{-\frac{t}{\tau }}$$

Among them, $V_{0}$ is the initial potential, and τ is the time constant.

The smaller the τ, the larger the dissipation rate. The results show that BTO addition accelerates surface charge dissipation, with the fastest dissipation (smallest τ = 102.9 s), achieved at 3.7%. The fitting curves of the surface potential versus time for the six samples are shown in Supplementary Materials.

**Figure 6:**
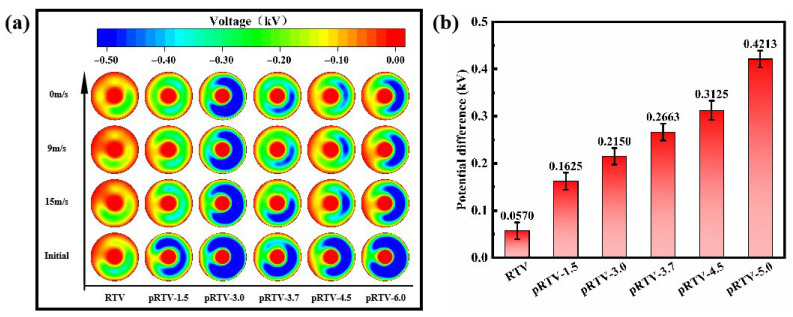
(**a**) Insulator surface voltage under wind disturbance. The experiment measured the potential change process on the insulator surface under slight disturbance using varying wind speeds (from 15 m/s to 9 m/s to 0 m/s). (**b**) The absolute value of the average potential difference on the insulator surface before and after the experiment reflects the influence of slight disturbance on the potential change in the insulator surface.

The aforementioned experimental method was used to polarize the surface of the insulator sample, with a voltage of DC 5 kV and a time of 15 min. Record its initial voltage. Then, blow one side of the insulator with wind at a speed of 15 m/s and 9 m/s in sequence to cause a slight disturbance, and record the voltages twice. Finally, record the voltage after the disturbance stops, as shown in Figure 6a. As can be seen from Figure 6b, as the filler content increases, the rate of surface potential decrease increases significantly. This is because the introduction of piezoelectric BTO filler leads to an increase in physical defects inside the coating, which causes a decrease in the deep trap density and an increase in the shallow trap density inside the RTV [42]. The decrease in deep traps decreases the bound charges inside the RTV, and the increase in shallow traps increases the charge mobility, resulting in the surface charges being more likely to migrate to the inside. This can promote the dissipation of surface charges and effectively prevent the accumulation of surface charges. At the same time, the introduction of the piezoelectric effect will cause the coating to generate a transient local alternating electric field when the insulator undergoes a slight deformation. This electric field can affect the deep traps inside the RTV and provide the energy required for the charges to escape from the traps, thereby promoting the release and dissipation of bound charges and also promoting the migration of surface charges to the inside. Therefore, after comprehensive consideration, BTO with a mass fraction of 3.7% was selected as the optimal formula.

**Figure 7:**
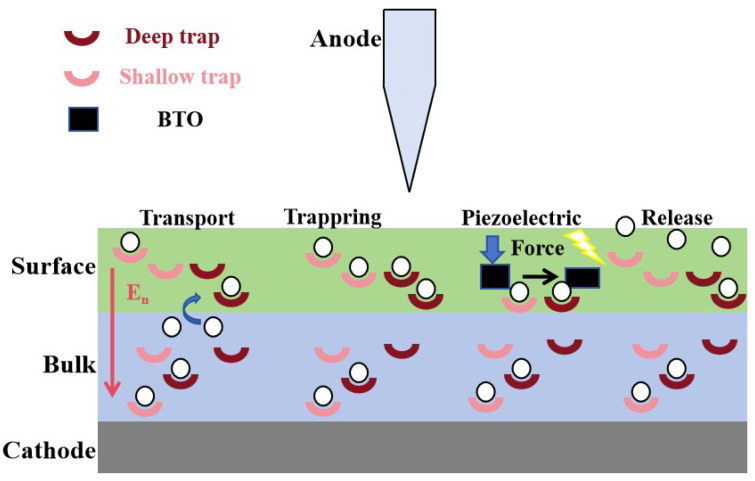
The action mechanism of surface charge dissipation on the pRTV coating includes charge transport, charges being captured by surface traps, the excitation of the piezoelectric effect by slight disturbances, and the piezoelectric effect causing the charges to break away from the trap capture.

The mechanism by which the coating accelerates the dissipation of surface charges is shown in Figure 7. Shallow and deep traps are distributed within the insulator and the coating [42,43]. When the needle electrode is energized, the electrons trapped by the deep and shallow traps inside the main body are released. Under the action of the normal electric field En, they are transported towards the electrode. After being transported to the surface, they are trapped by the shallow and deep traps on the surface, forming surface charges [44]. When the coating is slightly disturbed, the BTO fillers in the coating are subjected to force, and a potential difference is formed due to the piezoelectric effect. This potential difference will release the charges trapped by the deep and shallow traps, so the surface charges of the coating will dissipate rapidly under slight disturbance.

### 3.3. Pollution Accumulation Experiment

**Figure 8:**
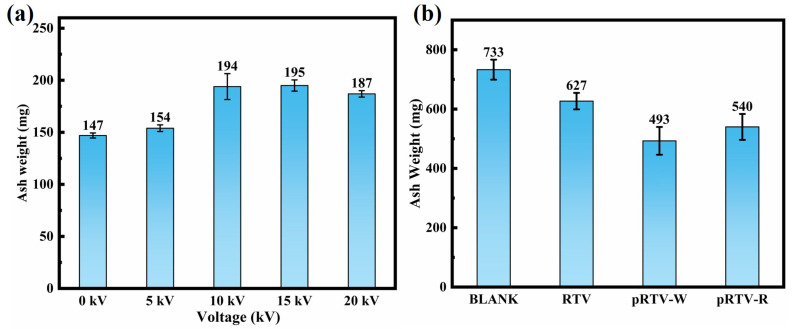
Results of insulator simulated pollution accumulation experiment (repeat 3 times, standard deviation). (**a**) Surface contamination of insulators (FXBW4-10/70) under five DC voltages (0 kV, 5 kV, 10 kV, 15 kV, 20 kV). (**b**) Surface pollution conditions of insulators with different coating layers under the condition of a polarization voltage of DC 10 kV.

To verify the anti-pollution performance of the coating on insulators, as shown in Figure S4a,b, we made a device for hanging insulators. This device was used to observe the surface contamination of the sample insulators, aiming to verify the anti-fouling performance of the piezoelectric coating. We studied the surface pollution accumulation of blank insulators under different voltages. As shown in Figure 8a, the results indicate that there is a positive correlation between the surface pollution of the insulators and the voltage, which is consistent with the conclusion put forward in Section 1. However, after the voltage exceeds DC 10 kV (the rated operating voltage of the insulators), the pollution accumulation no longer increases with the increase in the voltage. This is because the surface voltage on the insulator shed does not continuously increase with the polarization voltage. Instead, it reaches a saturation state after reaching a certain voltage. Therefore, the electric field force acting on the pollution particles will also reach a saturation state [45]. We applied the prepared coating on the sample insulators, a total of four insulators (namely Blank, RTV, pRTV-W, pRTV-R). In this experiment, we applied a voltage of DC 10 kV to the insulator, with a wind speed of 20 m/s. The pollutant was kaolin with a mass of 20 g. During the 1-hour experiment, the kaolin was evenly put into the test chamber. After the experiment, the residual kaolin attached to the surface of the insulator was collected and weighed. The results are shown in Figure 8b. It can be known from the results that the average NSDD of Blank in the three experiments is the largest, reaching 0.652 mg/${\mathrm{cm}}^{2}$. The sample coated with the RTV comes next, with a value of 0.557 mg/${\mathrm{cm}}^{2}$. The ash weights of pRTV-W and pRTV-R are smaller compared to the other two samples, with values of 0.438 mg/${\mathrm{cm}}^{2}$ and 0.480 mg/${\mathrm{cm}}^{2}$, respectively. This result indicates that the prepared samples have certain anti-fouling performance in the simulated environment. This further guides the research direction and prepares for the next simulation experiment under actual working conditions.

We applied the prepared coating on the sample insulators (Model: FXBW4-10/70). Two converter stations were selected for application simulation experiments in the southern coastal areas of China. The two converter stations are ±500 kV and ±800 kV, respectively: The ±500 kV converter station is adjacent to the industrial park, and the pollution content in the air is relatively high; the ±800 kV converter station is far from the industrial park, and the pollution content in the air is relatively low. See Figure S5 for the average temperature. Since the ESDD and NSDD are higher in coastal areas, flashovers are more likely to occur [46,47]. We chose to hang the sample insulators at the end of the year (November) and took samples once every three months, for a total of two samplings. It is worth noting that during the first sampling (February), due to the dry weather in autumn and winter, it had not rained in the areas where the two converter stations are located for a long time, so there was a large amount of pollution on the surface. During the second sampling (April), it was the plum rain season. There had been long-term rainfall at both converter stations within a week before the sampling, so there was less pollution on the surface. During sampling, for each insulator, we used four wet absorbent cotton balls to wipe each shed of the insulator and measured the ESDD and NSDD of each group of samples, respectively.

**Figure 9:**
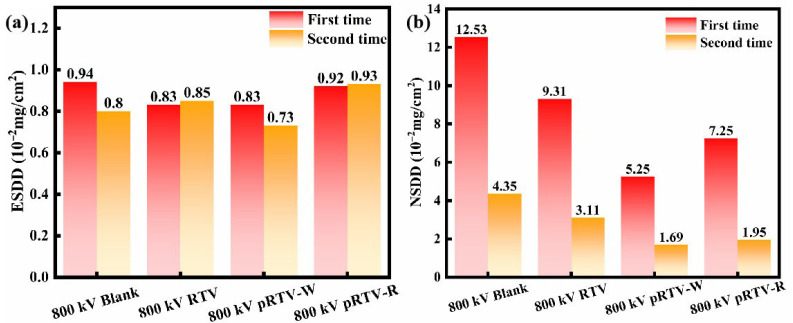
ESDD and NSDD of samples; the time intervals between the two samplings are both 3 months. The first sampling is conducted from October to January, and the second one is from January to April. The experiment was carried out under a DC field. (**a**) ESDD of samples in ±800 kV converter station, (**b**) NSDD of samples in ±800 kV converter station.

The measurement results of ESDD and NSDD are shown in Figure 9a,b and Figure S6a,b. We divided each insulator into four groups, and the final ESDD and NSDD values were averaged based on the surface area. From the results, it can be seen that since the ±500 kV converter station is located in an area with large dust, a large amount of dust has accumulated on the surface of the sample insulators, and the measured ESDD and NSDD are both relatively high. While the ±800 kV converter station is in an area with less dust, the ESDD and ash density measured in the experiment are both relatively low. The first sampling results of the ±500 kV converter station show that the NSDD on the surface of all sample insulators was very high. This is because when the first sampling was carried out, this area was farther from the coastline compared with the ±800 kV converter station, and there had been no rainfall for a long time, so the dust in the air settled on the surface of the insulators under the action of gravity. When the first sampling was carried out at the ±800 kV converter station, the NSDD on the surface of the sample insulators was significantly reduced compared with the results of the ±500 kV converter station, because this converter station is far from the road and belongs to an area with less traffic flow, so there is less dust in the air. When the second sampling was carried out at the ±500 kV converter station, both the ESDD and NSDD on its surface were significantly reduced. This is because the dust deposited on the surface of the insulators was carried away from the surface of the insulators by the rain [48]. Among them, the NSDD on the surface of the pRTV-R and pRTV-W samples was the lowest, which was reduced by 51.4% and 27.81%, respectively, compared with the blank sample, while that of the RTV sample was only reduced by 1.4%. The results show that the surface of the insulators coated with piezoelectric coating showed good self-cleaning performance. And the RTV sample insulators had less pollution deposited on the surface compared with the blank insulators, because the surface energy of the insulators coated with RTV paint was lower, and the pollution particles were more likely to be washed away by the rain. The second sampling at the ±800 kV converter station showed similar results to the second sampling at the ±500 kV converter station, pRTV-W and pRTV-R decreased by 55.17% and 61.49%, respectively, while the RTV sample only decreased by 28.51%. It is worth noting that the ESDD changes little after two samplings. This may be because the RTV coating itself has high hydrophobicity, which effectively reduces the dissolution and ionization of soluble salts. Therefore, external disturbances have little impact on it. The results further verified that the piezoelectric coating has excellent anti-pollution performance in the environment of high-voltage electric fields.

## 4. Conclusions

An RTV coating with piezoelectricity has been developed for UHV anti-fouling. Its dispersibility is enhanced through PDA-coated to increase its piezoelectric performance. Through piezoelectric and hydrophobic characterization, the optimal BTO addition amount was determined to be 3.7%. By increasing the attenuation rate of the surface potential of the insulator, its ability to attach charged contaminants is reduced. Through the wind simulation experiment, it is verified that it can promote the dissipation of surface charges under slight disturbances. Through the pollution accumulation simulation experiment, the anti-fouling effect of the coating under an external high voltage is verified. Finally, through the application experiment in the converter station, the anti-fouling accumulation effect of the coating is verified, and the NSDD is effectively reduced. Although the composite coating substantiates great anti-fouling performance, the operating conditions of pRTV are relatively limited. The weather resistance of the coating (such as in icing and sandstorm environments) is still needed further investigation for its practical engineering application. The anti-pollution accumulation coating developed in this study provides a new strategy for preventing pollution accumulation on the surface of insulators under ultra high DC fields and has application potential in the power field.

## Data Availability

The original contributions presented in this study are included in the article/Supplementary Material. Further inquiries can be directed to the corresponding author.

## Supplementary Materials

The following supporting information can be downloaded at: https://www.mdpi.com/article/10.3390/ma18235270/s1, Figure S1: (**a**) Insulator shed. (**b**) Sampling point; Figure S2: (**a**) Device diagram. (**b**) Schematic diagram; Figure S3: Hydrophobicity recovery curves of blank insulators and pRTV after plasma treatment; Figure S4: (**a**) ±500 kV sample rack. (**b**) ±800 kV sample rack; Figure S5: (**a**) Average temperature curve of ±500 kV converter station. (**b**) Average temperature curve of ±800 kV converter station; Figure S6: ESDD and NSDD of samles, the time intervals between the two samplings are both 3 months. The first sampling is conducted from October to January, and the second one is from January to April. (**a**) ESDD of samples in ±500 kV converter station, (**b**) NSDD of samples in ±500 kV converter station; Table S1: The initial potential ($V_{0}$) and time constant (τ) of the 6 samples; Table S2: Coating Physical and Chemical Properties Inspection Form; Table S3: Coating electrical performance Inspection Form.

## Abbreviations

The following abbreviations are used in this manuscript:

BTO	${\mathrm{BaTiO}}_{3}$
PDA	Pyridine dithioethylamine hydrochloride
PDMS	Polydimethylsiloxane
RTV	Room Temperature Vulcanizing
ESDD	Equivalent salt deposit density
NSDD	Non-soluble deposit density
PENG	Piezoelectric nanogenerator
