# Supplementary material for: Optimization of Anti-Fouling Piezoelectric Composite Coating for High-Voltage Insulators in Converter Stations
Source: Materials (Basel). 2025 Nov 21;18(23):5270. doi: 10.3390/ma18235270 (PMC12692940; doi:10.3390/ma18235270)
Supplement: Supplementary file 1 [file materials-18-05270-s001.zip › materials-3974533-supplementary.pdf]

Optimization of Anti-fouling Piezoelectric Composite Coating for High-voltage Insulators in Converter Stations

Yanwen Ouyang[1], Meng Chen[2,3], Siwei Pan[4], Qing Wang[4], Yihua Qian[4], Yuanyuan Li[1], Yong Liu[1], and Pengfei Fang[1,*]

[1]School of Physics and Technology, Wuhan University, Wuhan 430072, China
[2]Hubei Key Laboratory of Marine Electromagnetic Detection and Control, Hubei, China
[3]Wuhan Second Ship Design and Research Institute, Hubei, 430064, China
[4]Electric Power Research Institute of Guangdong Power Grid Co., Ltd., Guangzhou 510080, China

1 Introduction

Supplementary Materials

A Calculation processes of ESDD and NSDD

Wet absorbent cotton with distilled water (10 μS/cm), and then use the absorbent cotton to comprehensively wipe the surface of the insulator with accumulated contamination. After wiping off all the surface contamination, soak it in 500 mL of distilled water and stir it with a magnetic stirrer for 2 hours. After the contamination is completely dissolved, use a conductivity meter to measure the conductivity of the contamination solution and record the temperature of the contamination solution. The conductivity of the contaminated liquid measured by the conductivity meter should be converted to the value at 20 ℃. Combine the parameters of the insulator surface area and the amount of water used, and calculate the equivalent salt deposit density.

$$\sigma_{20} = \sigma_0[1 - b(\theta - 20)] \tag{S1}$$

θ is the solution temperature, ℃. σ_0 is the volume conductivity at a temperature of 0℃, S/m. σ_{20} is the volume conductivity at a temperature of 20℃. b is a factor depending on the temperature θ, which is determined by equation (S2).

$$b = \left(-3.2 \times 10^{-8}\right)\sigma^3 + \left(1.032 \times 10^{-4}\right)\theta + \left(3.55 \times 10^{-2}\right) \tag{S2}$$

Therefore, ESDD can be calculated according to equation (1) and equation (S2).

$$S_a = (5.71\sigma_{20})^{1.03} \tag{S3}$$

$$ESDD = S_a \bullet V|A \tag{S4}$$

Among them, S_a is the volume conductivity at a temperature of 20 ℃, S/m. $ESDD$ is the equivalent salt deposit density, mg/cm^2. V is the volume of distilled water, cm^3. A is the surface area of the insulator, cm^2.

After measuring the ESDD, suction filtration is performed on the pollution solution. By weighing the weight of the dried ash, the equation (S5) for the NSDD of the insulator is obtained.

$$NSDD = \frac{m_2 - m_1}{A} \tag{S5}$$

m_1 is the weight of the filter paper, mg. m_2 is the weight of the filter paper after suction filtration, mg. A is the surface area of the insulator, cm^2.

B Fitting curve of the decrease of the insulator surface potential with time

The fitting curve of the surface charge dissipation on the insulator usually follows the exponential decay law. The decay of the surface potential V(t) with time t is as follows:

$$V(t) = V_0 e^{-\frac{t}{\tau}} \tag{S6}$$

Among them, V_0 is the initial potential, and τ is the time constant.The initial potential(V_0) and time constant (τ) of the 6 curves are summarized in the table below:

Table S1The initial potential(V_0) and time constant (τ) of the 6 samples.

**Sample**	**V_0 (kV)**	**τ (s)**
RTV	0.101	224.7
pRTV-1.5	0.093	202.1
pRTV-3	0.095	198.1
pRTV-3.7	0.061	102.9
pRTV-4.5	0.053	185.3
pRTV-6	0.097	172.3

C Table

With the help of China Southern Power Grid, the physical, chemical and electrical properties of the coating were tested.

Table S2Coating Physical and Chemical Properties Inspection Form

**Test Item**	**Standard**	**Result**
Damp Heat Test	GB/T 1740-2007	No blistering/peeling/rusting after 1000h
Neutral Salt Spray Test	GB/T 1771-2007	No surface defects after 1000h
Thermal Cycling Test	GB 2423.22-2012	No defects after 25 cycles
Accelerated Aging Test	GB/T 14522-2008	No chalking/blistering
Adhesion Strength	GB/T 9286-1998	3.4 MPa
Self-Cleaning	DL/T 627-2018	Grade 1
Chemical Resistance	GB/T 529-2008	No peeling/wrinkling
Tear Strength	GB/T 528-2008	Type I dumbbell 3.0 MPa, 200%

Table S3Coating electrical performance Inspection Form

**Test Item**	**Standard**	**Result**
Pollution Flashover Withstand	DL/T 627-2018	2.18
Impulse Breakdown Voltage	GB/T 20642-2006 (28 kV test)	3 specimens passed no breakdown/damage
Dielectric Strength	GB/T 1408.1-2006	22.2 kV/mm
Tracking Resistance	GB/T 6553-2014	TMA 4.5
Surface Resistivity	GB/T 1692-2008	2.2×10^{12}

D Figure

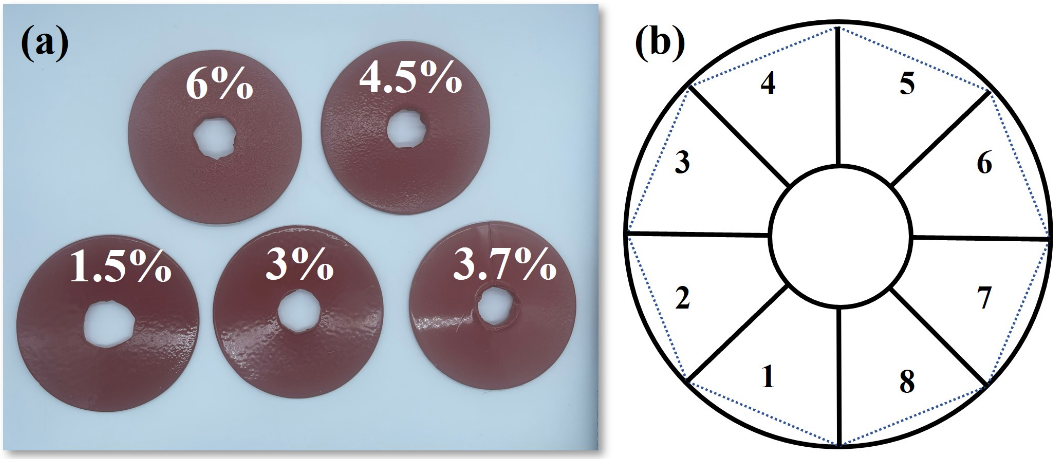

Figure S1. (**a**)Insulator shed. (**b**) Sampling point.

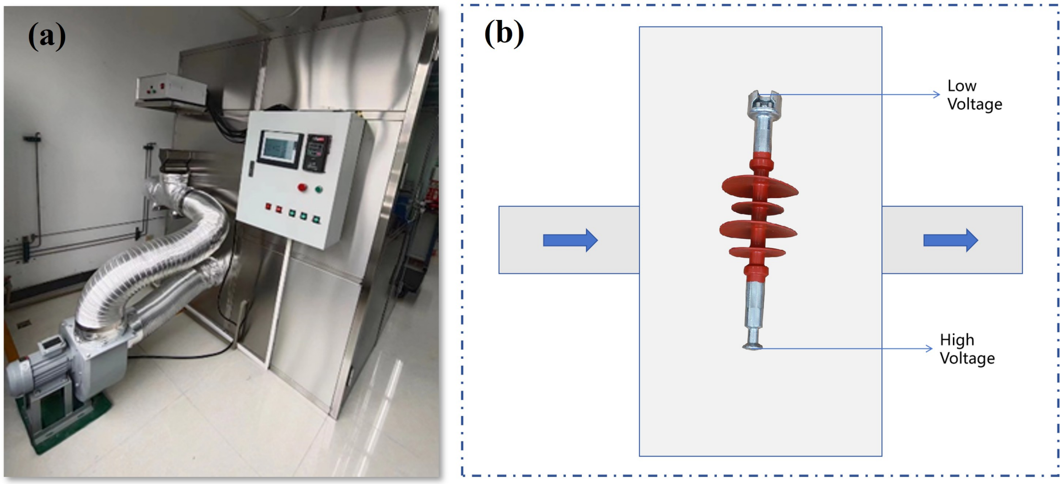

Figure S2. (**a**)Device diagram. (**b**) Schematic diagram.

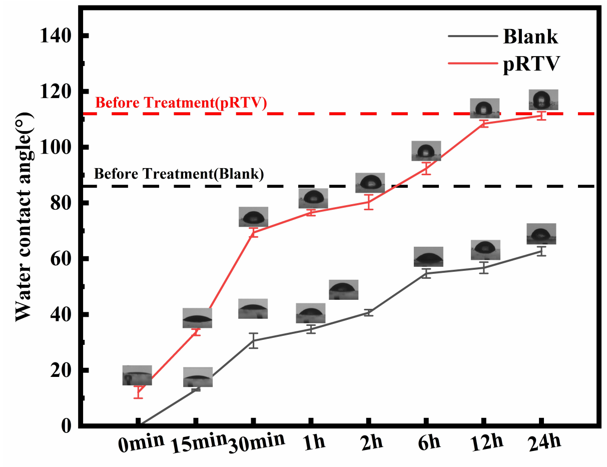

Figure S3. Hydrophobicity recovery curves of blank insulators and pRTV after plasma treatment

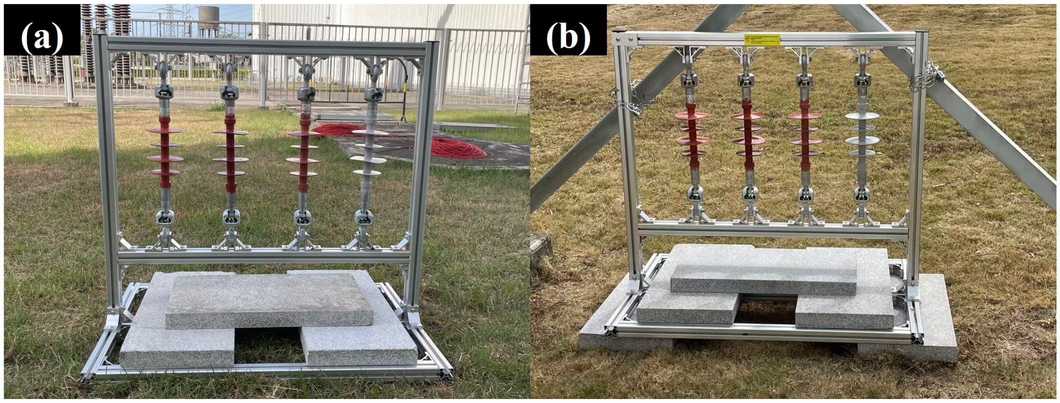

Figure S4. (**a**)±500 kV sample rack. (**b**) ±800 kV sample rack.

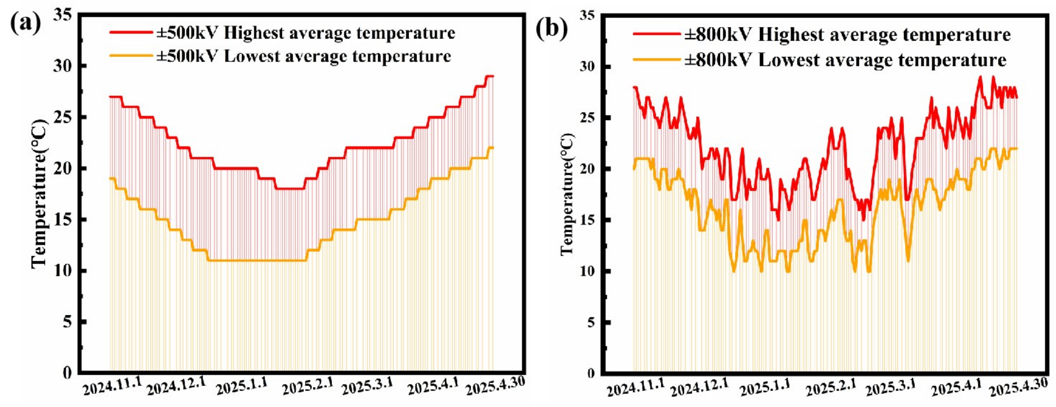

Figure S5. (**a**) Average temperature curve of ±500kV converter station. (**b**) Average temperature curve of ±800kV converter station.

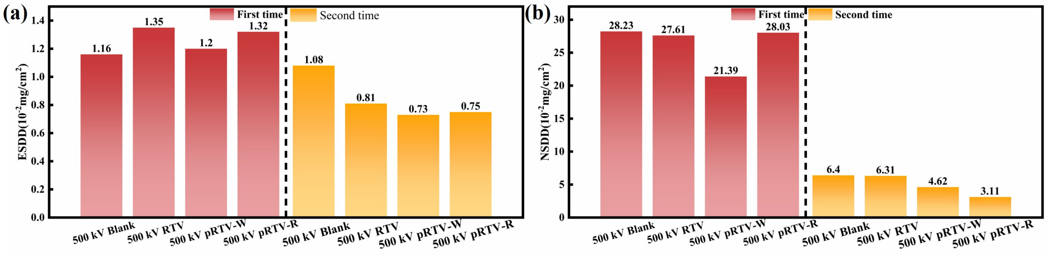

Figure S6. ESDD and NSDD of samles, the time intervals between the two samplings are both 3 months. The first sampling is conducted from October to January, and the second one is from January to April. (**a**) ESDD of samples in ±500 kV converter station, (**b**) NSDD of samples in ±500 kV converter station.
